# Machine Learning in the Analysis of the Mechanical Shredding Process of Polymer Recyclates

**DOI:** 10.3390/polym16131852

**Published:** 2024-06-28

**Authors:** Izabela Rojek, Marek Macko, Dariusz Mikołajewski

**Affiliations:** 1 Faculty of Computer Science, Kazimierz Wielki University, Chodkiewicza 30, 85-064 Bydgoszcz, Poland; dariusz.mikolajewski@ukw.edu.pl; 2 Faculty of Mechatronics, Kazimierz Wielki University, Chodkiewicza 30, 85-064 Bydgoszcz, Poland; mackomar@ukw.edu.pl

**Keywords:** artificial intelligence, machine learning, polymers, shredding, computational model

## Abstract

Artificial intelligence methods and techniques creatively support the processes of developing and improving methods for selecting shredders for the processing of polymer materials. This allows to optimize the fulfillment of selection criteria, which may include not only indicators related to shredding efficiency and recyclate quality but also energy consumption. The aim of this paper is to select methods of analysis based on artificial intelligence (AI) with independent rule extraction, i.e., data-based methods (machine learning—ML). This study took into account real data sets (feature matrix 1982 rows × 40 columns) describing the shredding process, including energy consumption used to optimize the parameters for the energy efficiency of the shredder. Each of the 1982 records in a .csv file (feature vector) has 40 numbers divided by commas. The data were divided into a learning set (70% of the data), a testing set (20% of the data), and a validation set (10% of the data). Cross-validation showed that the best model was LbfgsLogisticRegressionOva (0.9333). This promotes the development of the basis for an intelligent shredding methodology with a high level of innovation in the processing and recycling of polymer materials within the Industry 4.0 paradigm.

## 1. Introduction

Polymers have favorable properties: chemical resistance, weight reduction, simple shaping processes combined with additive manufacturing (3D printing), and recycling technology, which supports a comprehensive production process, new product designs, and material concepts, as well as product individualization [1,2,3]. The resulting increasing consumption of resources (including waste generation) and energy is difficult to meet. Solutions to this situation that fit into the Industry 4.0/5.0 paradigm are being sought [4,5,6].

Based on an analysis of the literature and the current state of science and technology in the field of comminution, it has been observed that there has been a growing interest among scientists and engineers in machine learning (ML)-based optimization of comminution technologies for polymeric materials with the expected grain-size curve of the comminution product envisaged for further use. ML methods and techniques are already supporting the development and improvement of shredder selection processes for processing polymeric materials. This makes it possible to optimize the fulfilment of selection criteria, which can include not only indicators related to shredding efficiency and recyclate quality, but also energy consumption in the shredding process. This fits into both the Industry 4.0 paradigm (automation and information and technical control at every stage of production) and Industry 5.0 (environment at its core and lifecycle analysis of materials and products in terms of their environmental and human impact) [7,8,9,10].

Open-loop recycling processes typically describe post-consumer plastic waste streams to create a specific input material (e.g., high-density polyethylene beverage bottle caps). It includes sorting, grinding, regranulation, and then forming (e.g., injection molding). Potential changes in the material throughout the recycling process can be reflected through melt flow rate (MFR), differential scanning calorimetry (DSC), and mechanical testing of different material states. The purity of the input stream is also important, depending on the form of collection and individual processing stages [11]. The use of modern material, including recycling, and innovative product design, taking into account its end-of-life, is crucial here to reduce (partially) or close (completely) the circulation of 3D printing materials [12]. Thermal, morphological, and mechanical properties of mixtures of virgin material and recycled material may vary depending on the proportions (1/3, 1/1/ and 3/1; however, the prevailing belief is that the content of recycled material is above 25–30%worsens the properties. Preliminary preparation of the recyclate is also important: grinding, sorting by size, washing (if any), and drying. The tests showed that adding polylactic acid (PLA) recyclate to the raw material did not significantly affect the thermal stability of 3D printed samples, and the original and processed PLA was subject todegradation at almost the same temperature. A more homogeneous structure was observed for samples made of 100% pure PLA and a more heterogeneous structure for PLA blends. Interestingly, the tensile strength of PLA blends increased after adding a larger amount of recycled material. Mechanical properties of 3D printed elementswith an admixture of recyclate are different from the properties of elements 3D printed solely from virgin material, which requires further research, modeling, and computational analysis, including in terms of predicting the properties of such products [13]. A variety of waste can be recycled, including those from car demolition, improving some of the properties of the primary materials depending on the recyclate content (both as fillers and/or modifiers) [14]. The research concerns both pure material and recyclate, their microstructures, increasing the interaction between them (also using additives such as resins), bending and impact strength, and dynamic-mechanical analysis of newly obtained materials. The most advanced research concerns the joining of mixtures with mixtures instead of the traditional joining of pure materials. This can improve mechanical properties, especially in the elastic modulus at room temperature and in impact strength [15]. This also applies to such advanced processes as tribocharging of shredded plastics made of poly(ethylene terephthalate) (PET) and high-density polyethylene (PE-HD), as well as factors affecting the accumulated electric charge and the effectiveness of electrostatic separation [16]. Research is being undertaken on the discrimination of mixtures of fine particles originating from the grinding of electronic equipment, both metals and plastics [17].

A great deal of attention is being paid to the management of polymer waste [18,19]. Recommendations made were that the amount of packaging recycled should increase to 80% of all packaging used and that 80% of this amount should be used as secondary raw material, with the result that 64% of the packaging used should be materially reused. The recovery rate of plastics in Europe reached about 55%, with 22.5% destined for reuse and 31.5% for energy purposes. Options to eliminate polymer plastics from the packaging industry have also been considered, but this would result in a worsening waste situation measured in terms of weight (up 40%), energy requirements (up 220%), costs (up 220%), and volume (up 260%). More versatile processes for raw material and energy use of waste are now being identified, which—after possible sorting—will allow further processing of all types of polymeric plastic waste. These processes will provide a measure of the level of environmental benefit and have advantages in this respect over traditional management methods, i.e., the manufacture of products from virgin raw materials and their storage after use. A number of management methods have been developed, of which material recycling and energy recycling are particularly noteworthy [18,19]. In each of these methods, the primary process is shredding.

Production of polymer plastics worldwide amounts to about 260 million tonnes per year, of which about 60 million tonnes are in Europe [20,21]. In the assortment structure of polymeric materials, polyolefins predominate with a share of more than 60% and an increasing trend in production. For example, in 2009, the share of various grades of polyethylene (29%) and polypropylene (19%) was dominant over polyvinyl chloride (11%), polystyrene (8%), polyethylene terephthalate (8%), and polyurethanes (7%). Considering the short lifespan of plastic products (especially packaging), the fulfillment of the sustainability policy, and the criteria of the environmental procedures implemented (e.g., LCA), the effective management of this plastic waste will have a significant impact. Already in the 1990s, the management of polymer plastic waste received considerable attention [22,23,24,25]. Recommendations made at the time were that the amount of packaging recycled should increase to 80% of all packaging used and that 80% of this amount should be used as secondary raw material, which implied that 64% of the packaging used should be materially reused. The recovery rate of plastics in Europe in 2004 reached approximately 53% (today 55%), with 26% going to reuse and 24% to energy (today 22.5% and 31.5%, respectively). At the time, options to eliminate polymer plastics from the packaging industry were considered, but, according to the author, this would have resulted in a worsening of the waste situation, measured in increases in weight (by 40%), energy requirements (by 220%), costs (by 220%), and volume (by 260%). Currently, more universal processes for raw material and energy utilization of waste are indicated, which—after possible sorting—will enable further processing of all types of polymeric plastic waste [22,23,24,25].

A number of management methods have been developed, of which material recycling and energy recycling are particularly noteworthy [23]. In each of these methods, the primary process is shredding [24,25,26]. It is estimated that up to 20% of the world’s plastics production is subject to recycled shredding as a result of poor product quality and 100% recycled shredding after the use process [27,28,29,30,31]. Due to the viscoelastic properties of the shredded plastics and the universal nature of the equipment used, shredding is a fairly energy-intensive process. The energy requirement for shredding is in the range of (20 ÷ 500) kW-h/tonne, representing 25 ÷ 50% of the total energy required for processing [32,33,34,35,36,37]. It is estimated that up to 8% of the world’s energy can be devoted to pelleting and agglomeration, and in mineral processing up to 70% of the total energy is devoted to grinding [38,39,40,41,42]. Considering the useful form of the product and the analysis of steps with a high energy intensity in polymer processing, it seems expedient to develop research methods in grinding engineering. The comminution process in this approach has not yet been comprehensively described.

ML techniques have proven to be very effective in optimizing various industrial processes, including the analysis and optimization of mechanical shredding processes for polymer recyclates. This is particularly true for tasks such as the following:

▪ Data collection: data can be collected both from sensors installed in the shredding equipment to monitor parameters such as temperature, pressure, motor power, and particle size distribution, as well as collected historical data on input materials and shredding conditions.
▪ Data pre-processing: pre-processing techniques such as normalization, outlier removal, and feature engineering are used to improve the quality of the dataset.
▪ Model training: various machine learning algorithms can be used to develop models that capture the relationship between input parameters (e.g., material properties, shredder settings) and output variables (e.g., particle size distribution, energy consumption). Supervised learning algorithms, such as regression, decision trees, random forests, or gradient enhancement, can be used to predict shredder outputs based on input parameters. Alternatively, unsupervised learning techniques such as clustering can help identify patterns in the data without labeled results.
▪ Feature selection and dimensionality reduction: techniques such as principal component analysis (PCA) or feature selection algorithms can help reduce the dimensionality of the data while retaining relevant information, improving model performance, and reducing computational complexity.
▪ Model validation and evaluation: e.g., cross-validation, in which the dataset is split into multiple subsets for training and testing, and evaluation metrics tailored to the specific goals of the fragmentation process analysis, such as mean absolute error or coefficient of determination, can be used to assess model performance.
▪ Model implementation and monitoring: real-time implementation of models in shredding processes can provide insights and recommendations for optimizing operations. Continuous monitoring of model performance and periodic updates based on new data can help maintain model accuracy and relevance over time.
▪ Optimization and control: ML models can be integrated with control systems to optimize shredding parameters in real time, aiming to achieve desired outcomes such as specific particle size distributions or energy efficiency targets. Reinforcement learning techniques can be used to iteratively adjust control parameters based on feedback [43,44,45,46,47,48,49,50].

The aim of this paper is to select methods of analysis based on artificial intelligence (AI) with independent rule extraction, i.e., data-based methods (ML). This promotes the development of a basis for intelligent shredding methodologies with a high level of innovation in the processing and recycling of polymeric materials within the Industry 4.0 paradigm. The novelty of this work is the combination of an observed research gap relevant to scientists, engineers, clinicians, and other end-users (local governments, the public) and the requirements of the Green Deal strategy with innovative ML technology to address them.

## 2. Materials and Methods

This manuscript explores the application of machine learning (ML) techniques to optimize the mechanical shredding process of polymer recyclates in order to increase energy efficiency and product quality. We have tried to ensure that all the results of the manuscript are reproducible based on the details provided in this section (both on the dataset and the methods used), as well as the supporting data.

### 2.1. Data Set

The study used data from research into the development of a method for selecting multi-edge shredders for processing polymer materials (PLA). PLA (polylactide, polylactic acid) is a polymer of natural origin produced from maize or sugar cane (it is a bioplastic). The characteristics of the PLA material used in the study are as follows:

▪ Tensile strength: 37 MPa;
▪ Tensile: 6%;
▪ Modulus of elasticity: 4 GPa;
▪ Density: 1.3 g/cm^3^;
▪ Melting point: 173 °C;
▪ Glass transition temperature: 60 °C [51,52,53].

**Figure 1:**
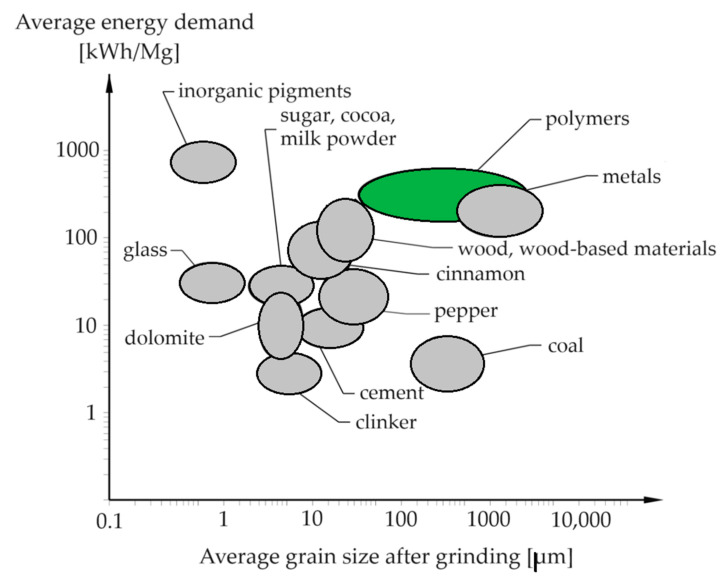
Average regranulate size and energy cost of shredding different materials [51,52,53].

The average regranulate size and energy cost of shredding different materials, especially polymers, which include PLA, are shown in Figure 1.

The aforementioned values are typical for PLA and allow for replication of the research as well as extension with other 3D printing materials.

**Figure 2:**
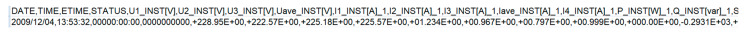
Part of the sample data set (one record from .csv file).

The main selection criteria are as follows:

▪ energy efficiency indices (Figure 2),
▪ improvement of shredding product quality.

This study took into account real data sets (feature matrix 1982 rows × 40 columns) describing the shredding process, including energy consumption used to optimize the parameters for the energy efficiency of the shredder (Figure 2). We also used oversampling to make the number of samples better meet the ML requirements. Each of the 1982 records in a .csv file (feature vector) has 40 numbers divided by commas. This dataset appears to be large enough to reflect the complexity of the destruction process and the number of parameters involved, as well as to increase the reliability of the ML models.

The main selection criterion is the improvement of the quality of the crushed product, hence there is a need to clarify how exactly this parameter was assessed. For technological purposes including the production and use of granular products obtained from grinding polymeric and biological materials, comparative indicators are used.These are parameters or features defined as properties of fine-grained materials, related to sets of grains. These indicators can be divided into three groups:

▪ Physical indicators defining the geometric features and physical properties of the grained material include dimensions, shapes of grains, distribution of grain sets, dimensions of control grains, external top or bottom grains, medium grains, percentage of the mass of the control grain, specific surface—kinetic, specific surface—static, pycnometric density, microhardness, compressive strength, tensile strength, brittle fracture strength, hygroscopicity, adsorption and absorption capacity, pyrophoricity, color, gloss, total pore volume, averagesize and distribution of pores, shape of pores, angle of internal friction, angle of static, and kinetic friction against metal, etc.;
▪ Chemical indicators describing the share of the basic component and other ingredients, the share of phase impurities (solid, liquid, and gas), the occurrence of oxide coatings, corrosion resistance, chemical activity, electro-chemical, catalytic ability, and toxicity, etc.

Technological indicators determining degrees of fragmentation (limital, average, n-percentage), degree of surface increase, susceptibility to grinding, susceptibility to agglomeration, water share, total efficiency, efficiency of the useful class, specific energy demand, efficiency index (effectiveness) grinding process, flowability, compactibility, compressibility, and sinterability, etc.

**Figure 3:**
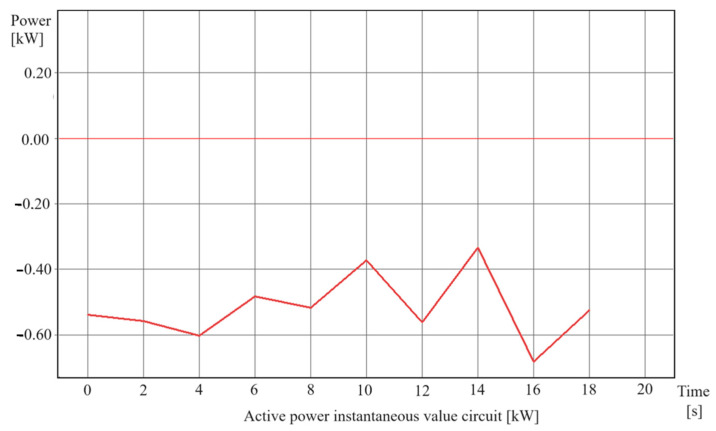

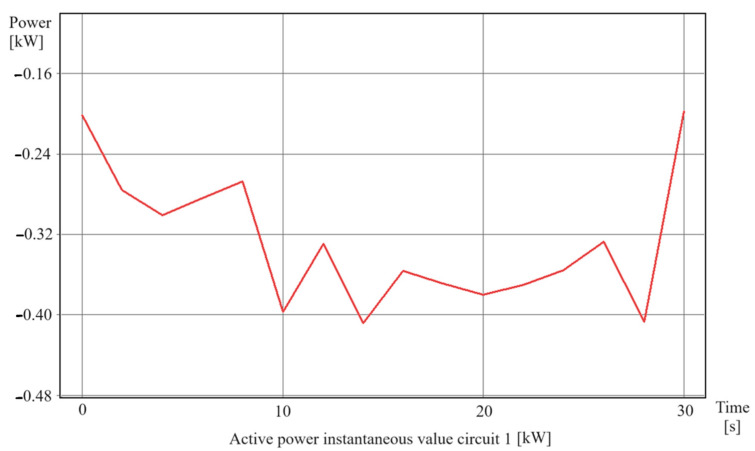
View of two sample data sets concerning active power.

Additional indicators are important for the user of the grinding product, including the physical and chemical properties of the material from which it is obtained, such as the structure of the material’s crystal lattice, fibrosis, isotropy or anisotropy, plasticity, the share of gases inside the grains and gases adsorbed on the surface of the grains, reactivity, adsorption capacity, type and number of network defects, microstructures, and grain surface condition (Figure 3). It should be noted that the quality indicators of grained materials depend on the type of material from which they are made and the method of their further use. Hence, the indicators for polymeric materials (for applications: multiple processing, fillers, dyes, fibers, seals, insulation, and others) are different than for biological raw materials (cereal grains), food (from flour to spices, pharmaceuticals), energy fuels (combustion in power boilers, gasification, processing into liquid fuels), animal feed and food, cosmetics, ceramic, metallic, composite biomaterials, composites, and brittle polymer materials, etc.

The data were divided into a learning set (70% of the data, 1388 records), a testing set (20% of the data, 396 records), and a validation set (10% of the data, 198 records).

### 2.2. Computational Methods

To date, artificial intelligence applications have been used to select the design of multi-disc shredders and to design/select the shape of the holes in quasi-cutting discs. It was assumed that the knowledge derived from the implementation of simulation research work, single-shredder tests, and laboratory studies could be codified and would facilitate the selection of a design solution depending on the properties of the feed material.

**Figure 4:**
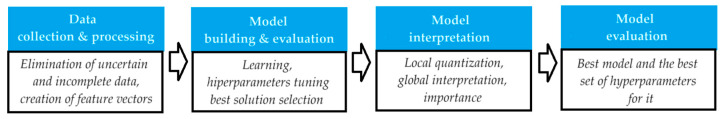
Flowchart of the machine learning mapping relationship established in this article.

In this article, as shown in Figure 4, we construct and interpret the dependencies of the mechanical grinding process of polymer raw materials to increase energy efficiency and product quality according to the following processes: (1) data collection and processing, (2) model building and evaluation, (3) model interpretation, and (4) verification on real data. First, we examined the important factors in the grinder affecting the energy consumption in the grinding process and the quality of the obtained granulate, using various algorithms and their hyperparameters (tuning hyperparameters allows to increase the accuracy of the algorithm) to identify the best model and the best set of hyperparameters for it. We established four sets of algorithms to verify the prediction model and mapping relationship, and the prediction results of the best of the above models (selected by cross-validation) are in very good agreement with the experimental results. In this way, we found a link between the improvement of grinder parameters and energy consumption and pellet quality from an ML perspective, which provides support for our further quantitative research.

As for the equipment and methods used in the study, at this stage of the research, in-house solutions written in the C# language in the Visual Studio 2022 environment including ML.NET (Microsoft, Microsoft, Oklahoma City, OK, USA) were used. ML.NET Automated ML (AutoML API) solutions available in the Visual Studio 2022 environment provide, above all, the ability to build your own pipeline with default trainers and search space configurations for classification and regression (binary classification, multiclass classification, regression).However, ML.NET supports many other scenarios, such as recommendations, forecasting, rankings, image and text classification, and sentence similarity.Additionally, for scenarios that do not have pre-configured search spaces and estimators, you can create your own to incorporate AutoML into your own scenarios dedicated to the solution (with support for both C# and .NET as well as your own APIs).

In the initial stage, dozens of available algorithms were examined for compatibility with the requirements of the research problem posed in this way. From these, the best five were then selected in terms of the specified criteria (accuracy, etc.) and subjected to hyperparameter tuning to optimize the results. Cross-validation of these five models ensured the selection of the optimal one among them.

The aforementioned test methodology was quick, simple, and relatively low-cost (in terms of both financial and computational cost) and is suitable for implementation and use in companies of all sizes and engineering staff capacities.

The code of the best solution can be downloaded both directly (as a piece of C# code for further use in a larger program) and as an API.

## 3. Results

When examining the algorithms, it was noted that there was a large variation not only in accuracy but also in learning time (by up to 1000%), which can be important for the selection of a particular algorithm (Table 1).Nevertheless, an accuracy of 90.00% has already been achieved at this stage, which seems sufficient to start tuning the hyperparameters in order to better fit the model to the application and the data.

Tuning the hyperparameters of the models resulted in an increase in accuracy of 3–5%, with a short learning time for the models (Table 2).

**Figure 5:**
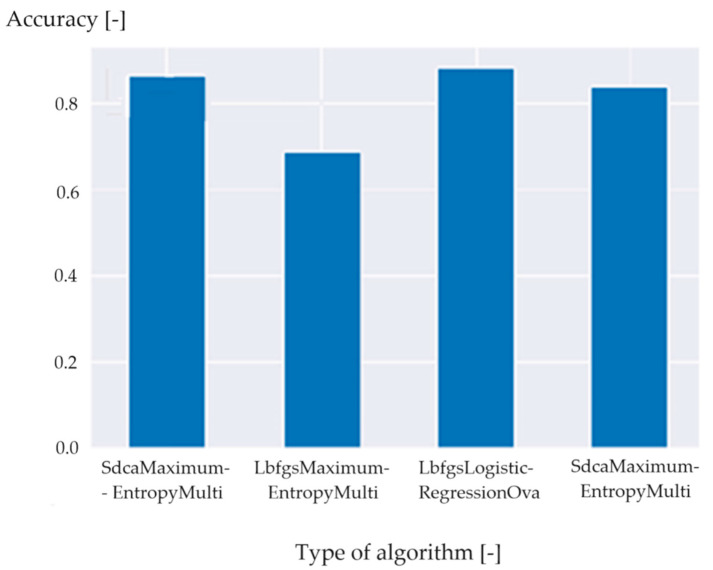
Cross-validation results. Algorithms one and four (counting from the left) are performed on different hyperparameters, hence the different results.

Cross-validation showed that the best model was LbfgsLogisticRegressionOva (Figure 5).

## 4. Discussion

The three main ways of dealing with polymer waste are as follows:

▪ reuse in unchanged form;
▪ recycling (material and/or energy);
▪ disposal (landfilling or incineration).

**Figure 6:**
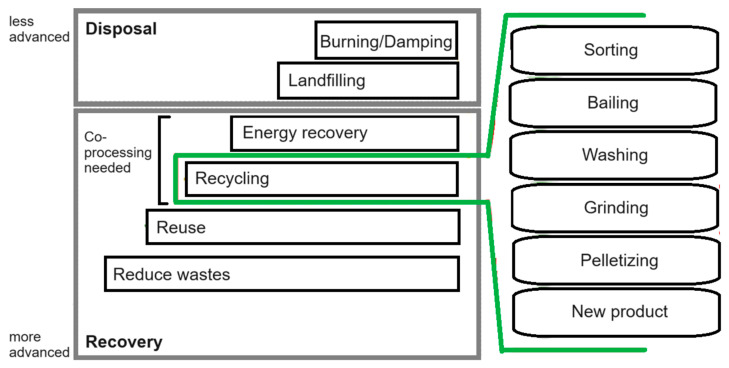
Place of recovery within dealing with polymer waste [41].

The first two of which are promoted as being more environmentally friendly. This also applies to multi-plastic or contaminated waste with structural changes resulting from contact with other materials, friction, temperature, and other process conditions, etc. Effective implementation of the above processes due to their commonness on an industrial scale requires the use of the following:

▪ preparatory operations (crushing, separating);
▪ dedicated installations and technologies AI/ML;

Of which the support helps in designing the above-mentioned processes in the context of sustainable development and circular economy (Figure 6) [54].

A critical review of six key bibliometric databases with the keywords ‘fragmentation’, ‘artificial intelligence’, and ‘machine learning’ did not return any results. This indicates not only the novelty of the topic of the application of ML in shredding but also the need for increased efforts by engineers and interdisciplinary teams (mechanical engineers, materials engineers, computer scientists, specialists in waste treatment and its environmental impact) so that innovative technical solutions such as ML can sufficiently support this fast-growing branch of Industry 4.0 and Industry 5.0.

Sustainable product development and optimal use of raw materials and resources (including water and energy) are becoming increasingly popular with industry, consumers, and decision-makers. In Germany, the simul+ Living Lab Sustainable Additive Manufacturing (SAMSax) has even been established to improve the durability of products by the following:

▪ designing products and services in terms of the possibility of life cycle analysis and recycling,
▪ selection of resource-efficient production processes,
▪ use of renewable materials,
▪ reducing energy consumption during use,
▪ open innovation process for current and potential/future stakeholders,
▪ increased acceptance and chances of implementation.

It is believed that an effective way to achieve the above-mentioned purpose leads through the following:

▪ waste upcycling, creating networks and active knowledge transfer via digital technologies, including ML/AI (Table 3),
▪ analysis and development of solutions within an integrated and coherent circular economy,
▪ testing and evaluation of innovations in a real environment, including in small and medium-sized enterprises.
▪ The most promising raw materials include:
▪ paper and wood dust,
▪ sand,
▪ harvest residues [20].

**Table 3:** Place of ML within shredding [54].

Factors	
Internal	External
Material: size, shape, density, strength, hardness (ML-based material assessment and preparation)	Environment: Temperature, humidity, pressure (ML-based environment compensation)
Machine: design, number of cutting edges, shape of cutting edges, movement direction (ML-based machine adjustment)	
Process: duration, cutting velocity, number of cutting contacts (ML-based process optimization)	

Comparison of algorithms and their combinations for waste electrical and electronic equipment (WEEE) showed characterization accuracy greater than 95%, which allows for the following:

▪ improving the efficiency of waste disposal from electrical and electronic products introduced to the market every year,
▪ recovery of the content of key metals contained in the above-mentioned products (including mixtures of fine particles from WEEE grinding).

Despite the fundamental difficulties resulting from the following:

▪ high variability of materials used in the production of electrical and electronic equipment,
▪ a complex separation process requiring sensors and flexible AI/ML systems that are not currently used in recycling plants [17,55,56,57].

### 4.1. Discussion of the Results Obtained

Our study showed the possibility of standardizing the selection methodology based on determining the unit energy consumption to obtain a product with a given processing size, related to the type and form of the material. There is a need to extend research to materials other than PLA.

We proved the reliability of the ML model, and the prediction results of the ML model were basically consistent with all of the experimental results and hada relative error of less than 7%. Our research can accelerate the rational design and monitoring of the development of efficient grinders. As part of the study, a mapping model for improving grinder efficiency was constructed, and energy consumption and product (grant) quality were predicted. The results show that the relatively simple SdcaMaximumEntropyMulti algorithm provides the best solution (the most accurate, with the smallest errors) in a relatively short computational time. It trains a linear model based on normalized input and output vectors. It predicts the target using a maximum-entropy multi-class classifier.The trained model generates class probabilities. The model demonstrated improved prediction and generalization ability even with relatively little information about the representation of key structural features.

As we mentioned above, we did not find equivalents of our studies in the literature for comparison. Considering the results of other studies on the optimization and control of PLA shredders (including single-shot), it is noteworthy that so far most solutions have lacked the AI/ML component, and the result of modeling the actual shredder system with an accuracy of at least 90.00% is considered very good. For the aforementioned reasons, our results (93.33%) are relatively high and allow the development of research results to be applied in industrial practice. It seems that further development of the proposed method will result in an increase in the above values, but this depends on two factors:

▪ the accuracy of the description of the polymer feature vector/matrix,
▪ the matching of the best ML algorithm (including the transition to deep learning—DL).

### 4.2. Limitations of Current Studies

While ML offers numerous advantages in analyzing the mechanical shredding process of polymer recyclates, it also has several limitations:

▪ Data quality and quantity: In some cases, obtaining sufficient and high-quality data on the mechanical shredding process, including variables such as material properties, shredder settings, and process results, can be challenging. Limited or biased data can lead to inaccurate models and unreliable predictions.
▪ Complexity of the shredding process: Mechanical shredding processes for polymer recyclates can be very complex and involve various physical and chemical interactions. ML models can struggle to capture the full complexity of these processes, especially if important factors are not well understood or difficult to measure.
▪ Interpretability: Many ML/DL models are often considered black boxes, meaning that their inner workings are not easily interpretable by humans. In industries where interpretability is critical to decision-making, such as manufacturing, this lack of transparency can be a significant limitation.
▪ Generalization: ML models trained on specific data sets may have difficulty generalizing to new or unfamiliar data. Changes in materials, equipment, and operating conditions can lead to model degradation or failure if not properly accounted for during training.
▪ Over- and under-fitting: ML models are prone to over-fitting (capturing noise in the training data) or under-fitting (not capturing underlying patterns). Balancing model complexity and generalizability is essential to mitigate these issues.
▪ Concerns about bias: ML models may inadvertently perpetuate biases present in the data used for training, which can lead to unfair or environmentally unsustainable recommendations/decisions.
▪ Dynamic nature of ML processes: they may exhibit dynamic behavior, with parameters changing over time or in response to external factors; for the above reasons, static machine learning models may struggle to adapt to such dynamic environments without continuous retraining or adaptive mechanisms.
▪ Computational resources: training complex ML/DL models can require significant computational resources in terms of processing power and memory, which can present challenges in implementing and maintaining such models [58,59,60].

### 4.3. Directions for Further Research

By pursuing the research directions described below, the application of ML in the analysis of mechanical shredding processes for polymer recyclates can develop, leading to more reliable models, a better understanding of the process, and sustainable recycling practices:

▪ Data augmentation and simulation: Developing techniques to augment limited shredding process data through simulation or synthetic data generation. This may include creating realistic virtual environments to simulate shredding processes or generating synthetic data using physics-based models to supplement experimental data.
▪ Multi-scale modeling: Exploring multi-scale modeling methods that can capture interactions between different length and time scales in the shredding process. This may include the integration of machine learning with computational fluid dynamics (CFD) or finite element analysis (FEA) to model macroscopic grinding behavior together with microscopic material properties.
▪ Dynamic and adaptive models: Developing machine learning models that can dynamically adapt to changes in the conditions of the grinding process in real time. This may include the use of techniques such as online learning or reinforcement learning to continuously update models based on incoming data and feedback from shredding equipment.
▪ Interpretable ML: This may include, for example, the development of model-independent interpretation techniques or the incorporation of domain knowledge constraints into model training to improve model transparency and reliability.
▪ Quantification and uncertainty propagation: This may include Bayesian methods, ensemble learning, or probabilistic modeling approaches to provide uncertainty estimates along with model predictions.
▪ Transfer learning and domain adaptation: Exploring transfer learning and domain adaptation techniques to exploit knowledge from related domains or processes where data may be more abundant; this includes pre-training models on similar tasks or domains before tuning them to chunk process data to improve model performance with limited data.
▪ Integration of ML models into real-time or near real-time process control systems: Adaptive process optimization, which may include the development of control strategies based on ML models or hybrid control architectures that combine machine learning models with traditional control algorithms.
▪ Industry/social implications: Exploring the environmental impact of ML model-based process optimization, addressing bias and fairness in decision-making, and ensuring transparency and accountability in model implementation.
▪ Joint research initiatives: Fostering collaboration between researchers (from different disciplines: mechanical engineering, computer science, materials engineering), economic stakeholders, and regulators to establish common datasets (and rules for organizing and sharing them), practice patterns (including data processing and imaging) and best practices for the application of ML in grinding process analysis. This could facilitate knowledge sharing, accelerate research progress, and ensure the relevance and applicability of research findings to real-world challenges [61,62,63,64,65,66,67,68,69].

The SWOT analysis (strengths, weaknesses, opportunities, and threats) showed the importance of the possible strengths of the proposed solution (Table 4). Weaknesses and threats are similar in all Industry 4.0 and Industry 5.0 systems and preparations should be made in this area before and during the introduction of innovative ML-based optimization. These may include the need for better computerization, automation, and robotization of the assessment of the environment, material, or shredding process as a whole.

The computational approach increases the efficiency of the monitoring system and the rational use of the shredder’s resources (equipment, energy), allowing the focus to be on maximizing shredding efficiency and predictive maintenance. Comparing the results of successive predictions with the results of specific processes repeated for different cycles, materials, shapes, and (in some cases) interchangeable shredding tools will result in more accurate, tailored, and predictive results.

Semi-automatic and automatic collection of large amounts of data from the grinding process should become natural. This will not only allow for a better understanding of the grinding mechanisms themselves but also take into account other factors (not taken into account so far). The influence of local factors (humidity or dustiness of the environment, etc.) may affect the quality of grinding products. The above approach may be one of the systemic ways to improve the quality of products and reduce the environmental impact of Industry 4.0/5.0.

The described ML system should be technologically and legally related to the entire industry operation system, including in the areas of systematic, cyclical testing of devices and entire production lines, automatic availability of collected data, and the possibility of comparing them in the light of the life cycle analysis of products and devices (machines, production lines) and their carbon footprint. This will allow us to better investigate the effects of recycling and their impact on the environment. The proposed ML-based solution will allow for better use of the above-mentioned opportunities.

It should be assumed that environmental design methods will continue to be developed dynamically. For this reason, it will be a necessary condition in the future to have the ability to implement and follow these procedures. The selection of structural, kinematic, and material parameters, carried out in accordance with the adopted method, guarantees high energy efficiency. It is important to take into account grinding utility indicators, the inclusion of which contributes to the construction of a multi-edge grinding model, in terms of identifying high energy efficiency and a product with the expected grain-size curve.

It is necessary to develop a methodology for selecting multi-edge shredders for processing polymeric materials based on modern IT tools (especially AI/ML-based) for faster creation and expansion of a universal shredding knowledge base, built and perhaps further expanded based on a framework expert system. Based on literature reviews and our study, the research problem is well recognized and its satisfactory solutions are known, but data-based models (ML-based) should be further improved, which would enable relatively quick obtaining and optimization of such solutions computationally.

It is worth noting that such an optimization algorithm for the selection of multi-edge shredders should take into account modern design methods and scientific research/analysis, hence it is necessary to ensure cyclical improvement and updating.

## 5. Conclusions

The introduction of active control systems based on ML is crucial for the automation and computerization of the grinding process. Their primary task is to minimize the negative impact on the environment (by reducing the consumption of energy and raw materials, the amount of waste, dust, and gas emissions) while maximizing the effects (improving efficiency in obtaining a product of the required size).As ML-based shredder solutions are developed, the greatest challenge will be reducing the energy consumption of the entire process.

This paper shows that it is possible to adapt modern ML-based design methods to find innovative design solutions for the shredder space. By using ML in the analysis of mechanical shredding processes for polymer recyclates, the industry can improve efficiency, reduce waste, and improve the quality of recycled materials, contributing to sustainability and profitability goals.

The sustainability of materials in blends of recycled post-consumer plastics requires additional research not only on composition and various methods (including hybrid), followed by the comparison of costs and printability of blends (including dimensional accuracy of prints) and life cycle assessment (LCA).This will allow you to decide on the optimal mix (mechanical, ecological properties) in terms of material, economic, and sustainability benefits.

## Figures and Tables

**Table 1:** The results of searching the set of 34 algorithms for a match to the dataset at hand.

Algorithm	MicroAccuracy	MacroAccuracy	Duration (Related)
SdcaMaximumEntropyMulti	0.5167	0.5500	4.0
FastTreeOva	0.0900	0.0750	4.9
SdcaMaximumEntropyMulti	0.0900	0.0750	18,6
LbfgsMaximumEntropyMulti	0.6600	0.7083	1,2
SdcaLogisticRegressionOva	0.1033	0.1083	21.6
LbfgsLogisticRegressionOva	0.6600	0.7083	2.0
FastForestOva	0.6000	0.6000	8.4
LightGbmMulti	0.6712	0.6933	1.9
FastTreeOva	0.0900	0.0750	8.2
SdcaMaximumEntropyMulti	0.8667	0.8667	2.2
LbfgsMaximumEntropyMulti	0.6600	0.7083	1.1
SdcaMaximumEntropyMulti	0.8667	0.8667	2.2
SdcaLogisticRegressionOva	0.0333	0.0333	13.3
LbfgsLogisticRegressionOva	0.9000	0.9000	2.4
LbfgsMaximumEntropyMulti	0.7600	0.7600	1.2

Note: the two algorithms with the same name differ in their hyperparameters and results.

**Table 2:** The results of the best five algorithms after tuning of hyperparameters.

Algorithm	MicroAccuracy	MacroAccuracy
LbfgsLogisticRegressionOva	0.9213	0.9333
SdcaMaximumEntropyMulti	0.8799	0.8912
SdcaMaximumEntropyMulti	0.8667	0.8667
LbfgsMaximumEntropyMulti	0.6979	0.7238
LbfgsLogisticRegressionOva	0.6801	0.7182

Note: the two algorithms with the same name differ in their hyperparameters and results.

**Table 4:** SWOT analysis for ML-based shredding system.

Strengths	Weaknesses
IoT support 24/7 monitoring ML-based analysis and prediction Warnings and alerts Multipurpose use Automatization of data collection Relatively low cost per device/machine Intuitive use Individualized use	Limited number and quality of data sets to begin Lack of historical data sets Introduction requires educated specialists
Opportunities	Threats
Reduced workload toward optimization Early diagnosis Preventive intervention Easier testing Novel diagnostic methods Novel factors taken into consideration within processes Possibility of standardization Quick further development Part of bigger systems	Non-acceptance of AI/ML

## Data Availability

The datasets are partially available as Supplementary Material. The complete dataset is available from the authors upon request.

## Supplementary Materials

The following supporting information can be downloaded at: https://www.mdpi.com/article/10.3390/polym16131852/s1, supplementary_data_set_record.csv.
